# Bibliographical

**Published:** 1866-10

**Authors:** 


### BIBLIOGRAPHICAL.

*A Treatise on Military Surgery and Hygiene.* By FRANK HASTINGS HAMILTON, M. D., late Lieutenant-Colonel, Med. Insp. U. S. A., Prof. of Mil. Surgery and Hygiene, and of Fractures and Dislocations, in Bellevue Medical College, etc., etc. Illustrated with 127 Engravings. New York. Bailliere, Brothers, 520 Broadway. 1865.

This work was, several weeks since, transmitted by the publishers to a party, supposed to be one of the editors of this Journal, who has handed it to us only recently. Hence our failure to notice it at an earlier date.

It is a valuable hand-book for the student of Military Surgery, and a creditable monument of the industry and scientific attainments of its distinguished author.

The chapters relating to the general hygiene of troops, bivouac, accommodation of troops in tents, barracks, billets, etc., hospitals, preparations for the field, hygienic management of troops upon the march, and the conveyance of sick and wounded soldiers, are full of interest, and we know of no work, in which the subjects are so particularly treated.

The chapters on gunshot wounds, in general, and severally of the head, face and neck, thorax, abdomen and of the male organs of generation; on punctured and incised wounds of the thorax and abdomen; on gunshot fractures, amputations, exsections, arrow-wounds, traumatic, and hospital, and dry gangrene, tetanus, scorbutus, and anæsthetics, furnish a valuable compendium of practical information upon these subjects, which we commend to the attention of the earnest student.

The typography, paper and binding are highly creditable to the publishers of the work.

